# Epidemiology and Risk Factors of Table-Tennis-Related Injuries: Findings from a Scoping Review of the Literature

**DOI:** 10.3390/medicina58050572

**Published:** 2022-04-21

**Authors:** Carlo Biz, Luca Puce, Maamer Slimani, Paul Salamh, Wissem Dhahbi, Nicola Luigi Bragazzi, Pietro Ruggieri

**Affiliations:** 1 Department of Surgery, Oncology and Gastroenterology (DiSCOG), University of Padova, 35128 Padova, Italy; pietro.ruggieri@unipd.it; 2 Department of Neuroscience, Rehabilitation, Ophthalmology, Genetics, Maternal and Child Health (DINOGMI), University of Genoa, 16132 Genoa, Italy; luca1puce@gmail.com (L.P.); maamer2011@hotmail.fr (M.S.); 3 Krannert School of Physical Therapy, College of Health Sciences, University of Indianapolis, Indianapolis, IN 46227, USA; salamhp@uindy.edu; 4 Tunisian Research Laboratory “Sport Performance Optimisation”, National Center of Medicine and Science in Sports (CNMSS), Tunis 1004, Tunisia; wissem.dhahbi@gmail.com; 5 Training Department, Qatar Police College, Doha 7157, Qatar; 6 Laboratory for Industrial and Applied Mathematics (LIAM), Department of Mathematics and Statistics, York University, Toronto, ON M3J 1P3, Canada; robertobragazzi@gmail.com

**Keywords:** table tennis, sports trauma, tenosynovitis, strains, scoping review

## Abstract

*Background and Objectives:* Table tennis represents one of the fastest ball games in the world and, as such, is characterized by unique physiological demands. Despite its popularity, there is a dearth of data related to table-tennis-related risk factors and injuries. Therefore, the present review was conducted to fill in this gap of knowledge. *Material and Methods:* The present review was designed as a scoping review. Eleven online databases were searched with no language/date limitations. *Results:* Forty-two investigations were retained in the present review. These studies indicated that tenosynovitis, benign muscle injuries, strains, and sprains were the most common injury types. In order, the most commonly affected anatomical regions were the lower limb, shoulder, spine, knee, upper limb, and trunk. When comparing the injury occurrence between training and competition, the results were contradictory. National/international athletes had higher indices of injury than regional players, even though other investigations failed to replicate such findings. According to some scholars, there was a difference between female and male athletes: in females, more injuries involved the upper limbs when compared to men who had more injuries to the lower limbs, while other studies did not find differences in terms of gender. *Conclusions:* Table tennis is generally considered at lower risk for injuries than other sports. However, the present scoping review showed that injuries can occur and affect a variety of anatomic regions. Sports scientists/physicians could utilize the information contained in the current review for devising ad hoc programs to adopt an effective/appropriate prevention strategy and to monitor table tennis players’ training load and to achieve maximal fitness, as these will reduce the risk of injuries. However, most of the studies included in our scoping review are methodologically weak or of low-to-moderate evidence, being anecdotal or clinical case reports/case series, warranting caution when interpreting our findings and, above all, further high-quality research in the field is urgently needed.

## 1. Introduction

Table tennis (TT) represents one of the fastest ball games in the world and, as such, is characterized by unique physiological demands [1,2,3,4], even though, according to Mitchell’s sports classification, it is a group 1B sport, with low static and moderate dynamic components [5].

Previous reviews have reported that TT requires moderate-to-high levels of aerobic/anaerobic power [2]. Poor anaerobic or aerobic capacity is of concern for the unconditioned, inexperienced competitor and, therefore, less technical skill and a lower physiological profile may be related to an increased risk of injury. Thus, prolonged periods of TT training each week and/or overtraining might increase such a risk.

Further, TT is characterized by highly skilled and coordinated movements of the hand/forearm acquired through repetitive exercises [6]. TT is a complex, challenging discipline that requires explosive speed, power, accuracy, reflexes, instantaneous decisions, and good management of the effects and the technique [7,8,9,10]. As such, players have to maintain high levels of prolonged focused attention and can be exposed to competitive anxiety [11,12] since competitions can last days [1].

For these reasons, the high psycho-physical demands of TT may increase the risk of injury. Several factors can predispose TT players to injuries and sports-related lesions. TT implies the adoption of a particular posture, namely flexed/semi-flexed knee and asymmetrically rotated trunk [13,14,15]. Barczyk-Pawelec et al. [13] evaluated the body posture in 40 TT players and 43 controls not practicing any sport. They found that TT players exhibited a kyphotic body posture, and they observed a statistically significant correlation between training experience in years and the amount of asymmetry of the inclination angle of the shoulder line. This finding has been confirmed and replicated by other investigations [14,15].

However, despite the popularity of this sport, to our knowledge, there are no reviews focusing on TT-related injuries. Identifying TT-related injuries may assist in preventing these injuries. Usually, TT is perceived as a safe sport, and injuries are generally managed with the RICER regime (R = rest the injured part; I = apply ice for 20 min every 2–3 h for the first 48 h; C = perform compression by applying a bandage; E = elevate/raise the affected part above the level of the heart; and, R = refer to a trained professional) [16].

The purpose of this scoping review was to systematically investigate and critically appraise the extant studies about TT-related risk factors and injuries. The synthesis contained in this review may inform sports scientists as well as coaches and provide useful information for future research and studies as well as for ad hoc training strategies and programs.

## 2. Materials and Methods

Given the breadth of our research questions, a scoping review was conducted to summarize data concerning TT-related risk factors and injuries. This emerging knowledge synthesis technique was preferred over other types of reviews (narrative or systematic ones) since the question was not narrow and focused on a specific topic but was, instead, quite open and inclusive, and we were more interested in width rather than depth. Relevant studies, major concepts, theories/theoretical frameworks, sources, and gaps in knowledge were identified, combined, and analyzed to map currently available evidence and to provide an overview of the extant scholarly research on the topics of the epidemiology of TT-related injuries and the related determinants/risk factors using a systematic and reproducible approach. Conclusions were based on available studies with suggestions for practical applications for strength and conditioning professionals as well as future investigations.

The present scoping review was carried out according to the “Preferred Reporting Items for Systematic Reviews and Meta-Analyses” (PRISMA) checklist [17] and its extension for scoping reviews (PRISMA-ScR) statement/guidelines [18]. The study was registered on the “Open Science Framework” (OSF) platform. Registration number: DOI 10.17605/OSF.IO/FQG7C.

Eleven databases were extensively searched from inception, looking for the following string of keywords with proper Boolean connectors: TT, sports-related injuries, sports-related lesion, and trauma. Truncated words with the wild card option and Medical Subject Headings (MeSH) terms were used when appropriate. The platform “Primo Central Ex Libris UNO per tutti” was used. No time restriction or language filter was applied. An update was carried out during the revision process of the article to include potentially relevant articles that were accepted and published in the interim period. The search strategy is detailed in Table 1.

Target journals—and in particular, the “*International Journal of TT Science*”, the official scientific journal of the organization International TT Federation, ITTF—were hand-searched.

In order to catch all relevant manuscripts, gray literature was also searched by means of Google Scholar. Theses and dissertations were also included. TT was searched also in different languages (for example: Tischtennis, tenis de mesa, tennis da tavolo, ping pong, among others). The studies were independently screened by two authors by reading study titles and abstracts during the first step and full texts during the second step for potential eligibility. The agreement rate was assessed using κ statistics and was solved by discussion; a third reviewer was involved when necessary.

Studies meeting the following “Population/Exposure/Comparison/Outcome(s)/Study design” (PECOS) criteria were considered for inclusion:

▸ P (population): male and female TT players at any age category and competitive level; in case the study also included other sports and disciplines, only data concerning TT players were extracted and retained in the present scoping review. If this was not possible (i.e., data were presented aggregated and were hard to disaggregate), the article was excluded;
▸ E (exposure): reporting sports-related injuries;
▸ C (comparison/comparator): between male and female TT players, various age categories, competitive levels, etc.;
▸ O: the type of injury, its epidemiology (incidence/prevalence rate), and determinants of injuries;
▸ S (study design): original peer-reviewed articles of any type (clinical case report, case series, observational study—incidence, prevalence or case-control investigation, randomized controlled trial, etc.) except for expert commentaries, editorials, and review articles, which were excluded but were scanned for potential articles to be included in the present scoping review;
▸ Languages: all languages available.

The risk of bias for all included studies was evaluated independently by two reviewers using the Joanna Briggs Institute (JBI)’s critical appraisal tools, which basically assess three major domains: (i) the trustworthiness, (ii) the relevance, and (iii) the results of included studies. Based on the specific investigation, the most appropriate JBI tool was selected, depending on whether the study was devised as a prevalence, incidence, case–control study, etc. The score was interpreted according to the JBI’s handbook: a percentage of “yes” in the range 100–70% was considered a low risk of bias, in the range 70–50% a moderate risk of bias, and in the range 50–0% a high risk of bias.

## 3. Results

From the search of the electronic databases, a total of 87 articles were found from PubMed/MEDLINE (*n* = 26), ProQuest Research Library (*n =* 14), ProQuest Health & Medical Complete (*n =* 14), ProQuest Science Journals (*n* = 6), Taylor & Francis Online—Journals (*n =* 5), BMJ Journals (BMJ Publishing Group) (*n =* 5), Informa—Taylor & Francis (CrossRef) (*n =* 5), Directory of Open Access Journals (DOAJ), (*n =* 4), ProQuest Health Management (*n =* 3), ProQuest Nursing & Allied Health Source (*n =* 3), and Wiley Online Library (*n =* 2).

After removing duplicates, 53 unique items were screened for potential inclusion. Forty-nine articles were added from the gray literature search. Reading titles and/or abstracts resulted in the elimination of 31 articles. Twenty-five studies were excluded for the following reasons: 10 investigations were focused on disabled TT players, for 7 articles it was not possible to distinguish TT players from other athletes (data is aggregated and not broken down for single-sport discipline), 3 studies did not meet the PECOS criteria (being focused on illnesses and not on sports-related injuries), 2 were not pertinent to the review question, 1 study was an expert opinion article, a further study contained little information, and finally, 1 investigation was a clinical commentary. A total of 41 studies [1,6,7,14,15,19,20,21,22,23,24,25,26,27,28,29,30,31,32,33,34,35,36,37,38,39,40,41,42,43,44,45,46,47,48,49,50,51,52,53,54] were included in the present review (Table 2 and Table 3).

**Figure 1:**
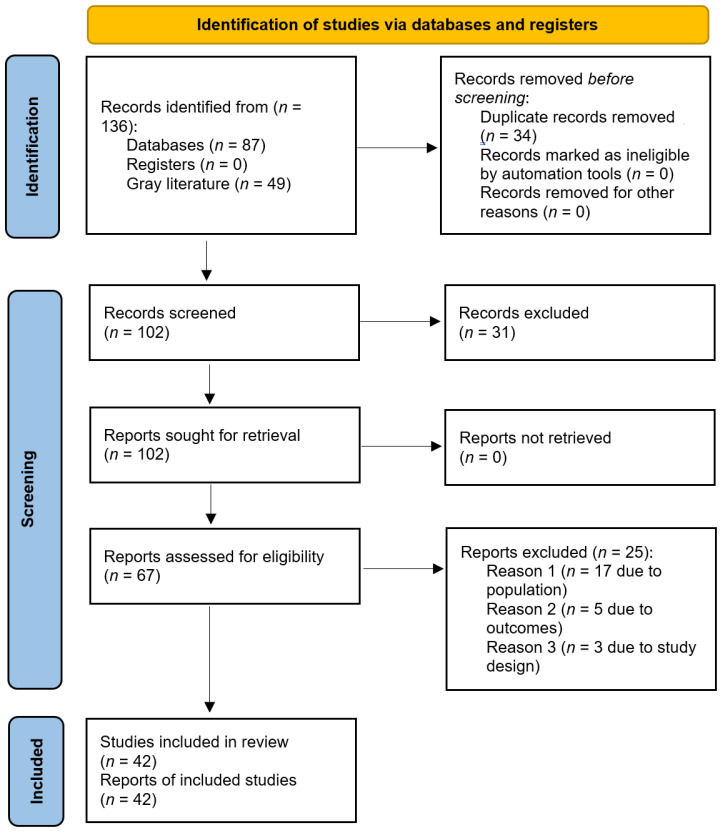
PRISMA flow-chart.

For further details about the identification and retention of the studies, the reader is referred to Figure 1.

Concerning the study design, 28 studies (66.7%) were devised as observational studies—in particular, 18 (64.3%) were incidence studies, 9 (32.1%) were prevalence studies, and 1 (3.6%) was a case–control study. Fourteen studies (33.3%) were designed as clinical case reports or case series. Finally, three (7.1%) were university theses/dissertations.

The sampling technique was mainly conducted by including consecutive participants, all athletes, or utilizing a convenience sample. Only in one case [20], athletes were randomly selected, and in one other article [7], a priori sample size estimation and power analysis were carried out.

Most studies focused on orthopedic injuries, while only a few considered eye injuries, cardiological risk factors, dental and facial injuries, dermatological lesions, neurological injuries, TT-related tumoral lesions, catastrophic injuries, and deaths. Finally, only one focused on risk injury perception and risk management strategies.

### 3.1. Risk of Bias Assessment

Concerning the risk of bias assessment, based on the JBI’s critical appraisal tools, only a few studies were deemed of high quality, the majority of the other studies included were methodologically poor/weak (low–moderate risk of bias) (Table 4).

More specifically, considering the observational studies, 39.3% and 53.6% of them presented moderate and high risk of bias, respectively, whilst 7.1% exhibited a low risk of bias.

### 3.2. Injury Rate

Two studies (4.8%) computed the overall injury rate among TT players [1,7]. As reported in the extant scholarly literature retained in the present scoping review, this rate is highly variable [1]. De la Torre Combarros et al. [1] (low risk of bias) observed that injury incidence in TT players rate was 2.8% (1.0% among males, 4.5% among females), and 66.6% of injuries occurred during the first day of the championship and 60% of lesions in the first hour of the afternoon. Correa-Mesa [7] (moderate risk of bias) found an injury rate of 44% among TT players. Males exhibited a 25% lower risk of developing TT-related injuries compared to females.

### 3.3. Orthopedic Injuries by Competitive Level, Gender, and Type of Contest (Training vs. Competition), and Location of Injuries

Twenty-two articles (52.4%) were focused on orthopedic injuries among TT players.

Due to rotational torques applied at the level of the knee joint, TT players may be predisposed to osteoarthritis of the knee. Rajabi et al. [15] (low risk of bias) found that 78.3% of the ex-elite TT players exhibited different radiographic signs of osteoarthritis versus 36.3% of controls. The Kellgren and Lawrence scale, which ranges from 0 to 4 and is used to quantitatively assess the radiographic severity of osteoarthritis, statistically differed between the two groups. Further, 68.2% of the ex-elite TT players reported symptoms of knee pain with the Western Ontario and McMaster University Osteoarthritis Index questionnaire versus 27.3% of controls. However, the stiffness and physical function categories of the questionnaire did not reveal any significant difference. On the other hand, 73.7% of the ex-elite athletes and 32% of the control group showed altered lower limb alignment (genu varum). The lower limb angulation also statistically differed between the two groups. In conclusion, radiographic evidence of osteoarthritis did not result in physical disability or knee stiffness. Shimazaki et al. [20] (moderate risk of bias) found that international and national athletes presented higher indices of injury (52.9%) than regional players (48.8%); the upper limbs (93.6%) and the trunk (87.5%) were the most affected sites. Finally, injuries were more likely to occur during training.

Correa-Mesa and Correa-Morales [21] (high risk of bias) found that the shoulder (17%), knee (16%), back (9.3%), and elbow (9.3%) were the most affected sites. The most prevalent type of injury was tendinopathy (38.2%) followed by benign muscle injuries (17.1%) and sprain lesions (10.9%). The most common diagnosis was rotator cuff syndrome (10.6%). Fong et al. [22] (moderate risk of bias) computed 240 sports-related lesions: 3 (1.3%) were ankle injuries and 3 (1.5%) were ligamentous sprains, while no fractures were detected. De la Torre Combarros et al. [1] (low risk of bias) found the injury incidence rate was 2.8% (1.0% among males, 4.5% among females), and 66.6% of injuries occurred during the first day of the championship and 60% of lesions during the first hour of the afternoon. The most serious lesion was a traumatic meniscus injury; other lesions affected the left knee joint, the internal malleolus of the right ankle, the rectus femoris, the supraspinatus of the dominant arm, and the thoracolumbar and the paravertebral muscles. Almost all injuries were acute, apart from a chronic one, namely the tendinosis of the supraspinatus. In conclusion, the most affected anatomic sites were, in order, lower limb, upper limb, and trunk. Di Carlo et al. [23] (high risk of bias) in Venezuela, recruited 26 players (18 males, 8 females) aged 10–50 years. Shoulder injury occurred in 84.6% of the sample, whereas lumbago was found in 100% of the individuals. Furthermore, supraspinatus and biceps tendinitis affected 63.6% and 36.4% of athletes, respectively.

Concerning risk factors, age was found to be the main determinant, with 54.6% of injured subjects being aged in the range of 10–20 years. Fernández Córdova and Barrios González [24] (high risk of bias) reported the following injuries: bursitis and synovitis (88%), lumbago (75%), tendinitis (25.21%), sacro-iliac joint pain (10.08%), tenosynovitis (8.40%), chondromalacia and insertion impairment (7.56%), and other injuries such as fasciitis, ganglionitis, capsulitis, subluxations, Osgood–Schlatter disease, herniated disc, and residual meniscus (7.56%). Risk factors were found to be international competitions and strenuous practice.

Furthermore, in a 10-year study, Majewski et al. [25] (high risk of bias) described 37 cases of knee joint traumas in 123,653 TT players (with a coefficient rate of 0.03).

Lo et al. [26] (high risk of bias) interviewed 373 physiotherapy students in the Hong Kong Polytechnic (242 males, 130 females) and reported 5 cases of shoulder impingement; in 3 athletes (60%), there was a frank clinical presentation of pain.

When comparing injury rates between male and female athletes and training and competition, Kondric et al. [27] (high risk of bias) recruited a sample of 83 top Slovenian athletes (29 players of TT, 39 of tennis, and 15 of badminton) and found that the most affected anatomic sites were the shoulder girdle (17.3%), spine (16.6%), ankle (15.8%), foot (10.1%), and wrist (12.2%). Most injuries involved muscle tissues and, to a lesser extent, joints and tendons/ligaments. According to the authors, injuries in TT are the consequences of “short, abrupt and extremely rapid movements, particularly in forehand strokes with no swing phase”. With the introduction of a bigger ball, the injury rate seems to have increased. Further, most injuries occurred both during a training session or a competition event at similar rates. Males and females were equally affected. The authors concluded that TT-related injuries are uncommon and occur at lower rates when compared to other sports disciplines in a statistically significant fashion.

Junge et al. [28] (moderate risk of bias) reviewed the injuries that occurred during the 2008 Summer Olympic Games and found that 9 athletes (5.2%) were injured with an estimated 2.6% of athletes reporting time-loss injuries. Five athletes (83.3%) reported injuries during training, only one (16.7%) during competition. Among the different sports disciplines, TT is the discipline with the highest injury rate occurring during training: one injury was a fracture and one a dislocation/rupture of tendon or ligament. The most hazardous sports disciplines were soccer, taekwondo, hockey, handball, weightlifting, and boxing, whereas TT was as safe as sailing, canoeing/kayaking, rowing, synchronized swimming, and diving, fencing, and swimming.

In contrast, Engebretsen et al. [29] (moderate risk of bias) found that out of 174 TT players (88 females, 86 males), 11 (6.3%) reported injuries. Seven (4.0%) and two (1.1%) injuries led to time loss ≥1 day and >7 days, respectively. Seven (70.0%) occurred during competition and three (30.0%) during training.

Linderoth [30] (high risk of bias) found differences between female and male athletes: in females, more injuries involved the upper limbs when compared to men who had more injuries to the lower limbs, possibly because women are generally weaker in the upper body and have hypermobile joints. Further risk factors for injuries can be warm-up and stretching performed poorly, as well as returning to sports practice immediately after the injury without a proper resting period.

Folorunso et al. [14] (high risk of bias) observed that 25% of TT-playing Nigerians complained of upper limb chronic pain. Further, Pieper et al. [31] reported three clinical cases (two males, one female) of impingement of the rotator cuff. Among the racket games, the sports that were more related to instability of the shoulder joint were handball (15 cases) and tennis (11 cases). TT was responsible for 4.5% of observed and treated shoulder injuries.

Nicolini et al. [32] described a clinical case of patellar tendinopathy in a female TT player treated at the Specialized Outpatient Clinics in São Paulo, Brazil, out of a series of 440 patients taking part in 33 different types of sports disciplines. Li [33] reported 19 clinical cases of De Quervain’s tenosynovitis. Other orthopedic injuries have been anecdotally reported, indicating they may be very rare among TT players. Pintore and Maffulli [34] reported the case of an osteochondritis dissecans of the elbow affecting the right lateral humeral condyle and clinically presenting as intra-articular loose bodies of the elbow. This lesion may be ascribed to repetitive valgus compressive stresses at the radio-capitellar joint. Ron et al. [35] described a case of simultaneous dislocation of both interphalangeal joints in one finger. Dufek et al. [36] reported a case of a stress fracture of the ulna in a 26-year-old professional TT player, probably related to the change of the intensity of training and of the TT racket.

Petschnig et al. [37] described a case of a stress fracture of the diaphyseal ulna in a 19-year-old female TT player, who also reported a history of secondary amenorrhea. The stress fracture was related to the vigorous movements carried out during the sport and repeated forearm flexor muscle activity due to overtraining. Conversely, no injuries were reported by Laoruengthana et al. [38] (high risk of bias), who concluded that TT is safe compared to sports disciplines such as handball, basketball, rugby, and football, which together accounted for 32% of all injuries, whereas no injuries were reported for other disciplines including shooting, dancing, and golf.

### 3.4. Dental and Facial Injuries

Three articles (7.1%) dealt with dental and facial injuries among TT players. Generally, TT is commonly perceived as a sports discipline characterized by a low rate of dental and facial injuries. Hill et al. [39] (moderate risk of bias) performed a cross-sectional study recruiting 130 patients from 21 different sports played in the Bradford area in the United Kingdom over five years. They found that miniature motorcycling and horseback riding were the most dangerous individual sports, while, as far as team sports are concerned, rugby, soccer, and cricket players reported the highest facial and dental injury rates.

Furthermore, de la Torre Combarros et al. [1] (low risk of bias) reported that facial injuries were the least common lesions among 10 types of injuries that occurred during the 2005 Spanish National Championships. Andrade et al. [40] (high risk of bias) reported no dental injuries among the five TT players who took part in the Pan American Games. The authors concluded that TT is at low risk for dental injuries; therefore, mouthguards are not necessary. Wrestling, boxing, karate, taekwondo, and field hockey should be considered at high risk, and basketball, team handball, soccer, water polo, baseball, judo, diving, and synchronized swimming at middle risk.

### 3.5. Dermatological Lesions

Patches and skin lesions have rarely been reported [41]. De la Torre Combarros et al. [1] (low risk of bias) reported 10 dermatological lesions overall, of which 2 (blisters affecting the fingers of the dominant hand in a female player) were due to TT.

### 3.6. Ocular Lesions

Blunt eye trauma is associated with many ball games, and TT players also seem to be predisposed to eye injuries. A systematic review performed by Barrell et al. [42] (moderate risk of bias) of the records of 118 patients treated at Southampton Eye Hospital during 1978–1979 for sports-related injuries revealed that even rare ocular lesions may require the use of eye protection. More in detail, 58 lesions (49.2%) were due to squash, 23 (19.5%) to badminton, 22 (18.6%) to football, 9 (7.6%) to tennis, 5 (4.2%) to cricket, and only 1 (0.8%) to TT.

Chandran [43] (moderate risk of bias) reported two cases of eye injuries among TT players in a period of 5 years in a series of 63 ocular lesions treated at the Eye Clinic of the University Hospital, Kuala Lumpur, Malaysia, involving a variety of sports (hockey, tennis, cricket, soccer, rugby, golf, squash, and other disciplines). Jeffers [44] (high risk of bias) reported two cases of eye injuries in two male TT players in the period 1984–1986 in a series of 203 ocular lesions. Kelly and Nolan [45] (moderate risk of bias) reported one case of a TT player with an eye injury out of 45 consecutive players who required admission to the hospital between 1 January 1979 and 31 December 1982.

### 3.7. Neurological Lesions and TT-Related Tumors

Information regarding neurological lesions and TT-related tumors is contained in anecdotal clinical case reports. For instance, Le Floch et al. [6] described four cases of focal task-specific dystonia, two involving professional international competitors. Copcu [51] described a case of one TT player who developed a very quickly grown lipoma on his right scapular area, probably a reaction to chronically minor traumas, especially to the scapular area.

As for cardiological risk factors, catastrophic injuries, and deaths, Fujiwara et al. [52] investigated 30 patients with sports-related acute myocardial infarction. They found that playing ball games was associated with this adverse event: 3 out of 30 played tennis or TT. Sudden death during TT play is anecdotal [53,54]. TT is safe with respect to other sports disciplines, such as horseback riding (98 deaths), air sports (92 deaths), motorsports (86 deaths), and mountaineering (74 deaths), which represent the most hazardous activities for adults, whereas the most hazardous activity for children is horseback riding (19 deaths), according to a comprehensive survey of sporting and leisure activities in England and Wales, carried out in the period 1982–1988.

### 3.8. Injury Risk Perception and Risk Management Strategies

Only one study assessed the knowledge and perception of injury risk among TT players. Chen-Liang and Wen-Guu [55] recruited 352 members of TT clubs in Taichung, Changhua, Chiayi, and Tainan via convenience sampling. Of these, 326 accepted to answer the questionnaire (92.6% participation rate, 7.4% dropout rate). They found that marital status influenced the perception of risk of injury in terms of “internal psychological factors”, but not in terms of “external environmental factors”. Further, “internal psychological factors”, “external environmental factors”, “self-cognitive factors”, “facility and equipment factors”, and “coach management factors” did not significantly correlate with one another.

Assessing injury risk perception as well as understanding risk factors and biomechanics of pain and injuries among TT athletes is of paramount importance to devise effective preventative training strategies. Evidence-based rehabilitation programs carried out in synergy with ad hoc training could allow pain- and injury-free sports activity, optimizing and enhancing the overall health benefits for TT athletes [56,57,58,59].

## 4. Discussion

TT is generally considered at lower risk for injuries than other sports [1,2,3,4,5]. However, injuries can occur and affect a variety of anatomic regions including the wrist, the elbow, the shoulders, the neck, upper back, and lower back, the hip, the ankle as well as muscles, ligaments, and tendons of these anatomical regions. Specifically, tenosynovitis, benign muscle injuries, strains, and sprains are the most commonly reported TT-related injury types [15,16,17,18,19,20,21,22,23,24,25,26,27,28,29,30,31,32,33,34,35].

In order, the most commonly affected anatomical regions are the lower limbs, the shoulders, the spine, the knees, the upper limbs, and the trunk [15,16,17,18,19,20,21,22,23,24,25,26,27,28,29,30,31,32,33,34,35]. TT-related injuries can be due to several parameters, such as poor warm-up and stretching, as well as poor training programs and overtraining. In the present scoping review, we have comprehensively scanned the extant scholarly literature. We have found an increasing interest in reporting and describing TT-related injuries over time, even though the range of injury rates is highly variable (depending on the study design, study population, country, and year). While there seems to be a general consensus that TT-related injuries occur at lower rates than with other sports disciplines, we found contrasting findings concerning their determinants.

For instance, when comparing the injury occurrence between training and competition, the results were contradictory. At the 2012 Olympic Games, higher injury occurrence during competition than during training was reported both in male and female TT players, whereas an opposite trend had been noticed during the 2008 Olympic Games [28,29]. Furthermore, national/international athletes presented higher indices of injury than regional players, even though other investigations failed to replicate this finding.

According to some scholars, there was a difference between female and male athletes: in females, more injuries involved the upper limbs when compared to men who had more injuries to the lower limbs, while other studies did not find differences in terms of gender. As such, the study of injury determinants in TT requires further high-quality research, also considering the overall low-to-moderate quality of the articles retained in the present scoping review.

### Strengths and Limitations of the Present Study

The strength of our scoping review consists of a rigorous, highly reproducible, and systematic literature search strategy, based on a solid rationale, clearly defined study inclusion/exclusion criteria and clearly defined primary and secondary endpoints/outcomes for evidence synthesis. Specifically, the search was without language/date limitations, and the risk of bias was assessed. In terms of the number of studies, we included 42 studies, and most of them focused on orthopedic injuries, which can help us extract data about the most commonly affected anatomical regions, injury types, and injury occurrence according to the type of contest, competitive level, and gender.

However, a number of limitations should be mentioned. We could not find in all the studies included injury rates during competitions reported stratified by gender and age, warranting that more data are urgently needed. Moreover, most of the studies included in our scoping review are methodologically weak or of low-to-moderate evidence, with some studies being anecdotal or clinical case reports/case series. Thus, few studies provided details of eye injuries, cardiological risk factors, dental and facial injuries, dermatological lesions, neurological injuries, TT-related tumoral lesions, and death. Only one study performed randomization of the sample and one computed a priori the power of the sampling technique. Therefore, these few published studies markedly understate the worldwide situation.

On the other hand, the present overview can serve to plan future higher-quality studies and epidemiological surveys to advance a field, which is generally overlooked, and to identify the best evidence-based training strategy aimed at preventing injuries among TT athletes.

## 5. Conclusions

The most frequently reported and studied TT-related injuries are orthopedic. Even if not precisely known, the rate of injuries seems to be low, especially when compared to other more dangerous sports. Generally speaking, TT is a relatively safe discipline [60,61], even though evidence collected and identified through the methodology of this study is of low-to-moderate quality based on the study designs and other intrinsic methodological deficiencies that we have underlined with our risk of bias assessment. Since most injuries occur during training, coaches should monitor the intensity of the training program. Overall, however, there is scant evidence about TT-related injuries. There is an urgent need for high-quality randomized studies, as also advocated by Steffen and Engebretsen [62]. A key approach for practitioners and sports physicians is to monitor TT players’ training load and to achieve maximal fitness, as these will reduce the risk of injuries.

## Figures and Tables

**Table 1:** Search strategy items and details.

Search Strategy Items	Search Strategy Details
Used keywords	“tennis table” AND (injuries OR lesion OR trauma OR traumatism OR risk factor)
Searched databases	PubMed/MEDLINE, ISI/WoS, ProQuest accessed via Ex libris platform at University of Genoa, Genoa, Italy
Inclusion criteria	Original article (of any type: case report, case series, observational study, randomized trial)
Exclusion criteria	Letter to editor, editorial, review articles
Time filter	None applied
Language filter	None applied
Target journals	*BMC Musculoskeletal Disorders; British Journal of Ophthalmology; British Journal of Sports Medicine; British Medical Journal; Cutis; Dental Traumatology; Injury; International Journal Of Sports Medicine; International Journal of Table Tennis Science; Journal of Physical Education, Recreation & Dance; Journal of Sports Sciences; Knee; Knee Surgery, Sports Traumatology, Arthroscopy; Revista Brasileira de Medicina do Esporte; Sports Medicine*

Abbreviations: WoS (Web of Science).

**Table 2:** Main features of the included studies (clinical case reports and case series are excluded and reported in a separate table).

Authors	Year	Country	Study Population	Age	Injury Rate	Risk Factors/Determinants
Anders Niixius et al. [19]	1976	Sweden	A series of 229 cases of Achilles tendon rupture	Nr	134 (121 m, 13 f) were due to sports activities, 5 (3.7%) of which were due to table tennis	Nr
Andrade et al. [40]	2010	Pan American Games (XV Pan Am) held in Rio de Janeiro, Brazil in July of 2007	5 table tennis players	Nr	0% (dental injuries)	Nr
Barrell et al. [42]	1981	United Kingdom	A series of 118 ocular lesions	Nr	1 case (0.8%) due to table tennis	Nr
Chandran [43]	1974	Malaysia	A series of 63 cases of ocular lesions	Nr	2 cases (3.2%) due to table tennis	Nr
Correa-Mesa and Correa-Morales [21]	2015	Colombia	50 athletes (9 f, 41 m) out of 52 athletes initially recruited	Nr	Shoulder (28%), knee (26%), and lumbosacral region (10%)	No risk factors found
Correa-Mesa [7]	2015	Colombia	178 athletes (141 m, 37 f)	21.77 y	44% (129 injuries), with rotator cuff syndrome being the most common disorder (10.6%). Shoulder (17%), knee (16%), back (9.3%) and elbow (9.3%) were the most affected sites. The most prevalent type of injury was tendinopathy (38.2%), followed by benign muscle injuries (17.1%) and sprain lesions (10.9%).	Higher BMI, female gender, older age, and longer training time were found to be significantly associated with an increased injury frequency.
de la Torre Combarros et al. [1]	2007	Spain	355 athletes (198 m, 157 f)	9–21 y	2.8%	66.6% during the first day of the championship, 60% during the first hour of the afternoon
Di Carlo et al. [23]	1997	Venezuela	26 athletes (18 m, 8 f)	10–50 y	Shoulder (84.6%), lumbago (100%), supraspinatus tendinitis (63.6%), biceps tendinitis (36.4%)	54.6% of injured subjects in the range 10–20 y
Engebretsen et al. [29]	2012	2012 Olympic Games	174 athletes (88 f, 86 m)	Nr	11 (6.3%) reported injuries. Seven (4.0%) and 2 (1.1%) injuries led to time loss ≥1 day and >7 days.	Seven (70.0%) occurred during the competition and 3 (30.0%) during training.
Fernández Córdova and Barrios González [24]	2008	Cuba	16 players (8 f, 8 m)	21 y, range 16–26 y	Bursitis and synovitis (88%), lumbago (75%), tendinitis (25.21%), sacrolumbalgia (10.08%), tenosynovitis (8.40%), chondromalacia and insertion impairment (7.56%), other injuries such as fasciitis, ganglionitis, capsulitis, subluxations, Osgood–Schlatter disease, herniated disc, and residual meniscus (7.56%)	International competitions, practice
Folorunso et al. [14]	2010	Nigeria	40 athletes (24 m, 16 f)	8 < 20 y, 32 > 20 y	25% (upper limb chronic pain)	Nr
Fong et al. [22]	2008	Hong Kong	A series of 240 ankle injuries out of a total of 1715 sports-related lesions and injuries	Nr	6 due to table tennis: 3 (1.3%) were ankle injuries, 3 (1.5%) ligamentous sprains	Lower ankle injury rate with respect to other sports, such as basketball or soccer.
Hill et al. [39]	1985	United Kingdom	130 patients with dental injuries related to 21 sports activities	Nr	Few table tennis-related injuries	Nr
Jeffers [44]	1988	USA	A series of 203 ocular lesions	Nr	2 cases due to table tennis	Nr
Junge et al. [28]	2009	Summer Olympic Games 2008	10,977 registered athletes	Nr	9 athletes (5.2%) were injured, with an estimated 2.6% of athletes reporting time-loss injuries. One injury was a fracture and 1 a dislocation/rupture of the tendon or ligament.	Five athletes (83.3%) reported injuries during training, only 1 (16.7%) during competition.
Kelly and Nolan [45]	1983	Ireland	A series of 45 consecutive patients (37 m, 8 f; 5 cases lost at follow-up)	25 y	1 case due to table tennis	Nr
Kondric et al. [27]	2011	Slovenia	83 athletes of which 29 table tennis players	19.52 ± 4.21 y	Shoulder girdle (17.3%), spine (16.6%), ankle (15.8%), foot (10.1%), and wrist (12.2%)	No risk factors found (training and competition reporting equal injury rates, also comparing females and males).
Laoruengthana et al. [38]	2009	37th Thailand National Games 2008 in Phitsanulok, Thailand	276 athletes (124 m, 152 f)	Nr	0%	Nr
Linderoth et al. [30]	2006	Sweden	85 athletes (only 44 taking part in the study)	Nr	79.5% (strain injuries 75%, upper limb 40%, lower extremity 35%, back injuries 22%, neck injuries 3%)	Poor warm-up and stretching, gender (women reporting more injuries to the upper extremity), poor resting after injuries
Lo et al. [26]	1990	Hong Kong	373 students (242 m, 130 f)	Nr	Shoulder impingement (1.3%)	Nr
Lovell et al. [46]	1995	Australia	A series of 189 chronic injuries	Nr	1 case of chronic groin pain	Nr
Majewski et al. [25]	2006	Switzerland	A 10-year survey of 123,653 athletes	Nr	37 cases of knee joint traumas	Nr
Rajabi et al. [15]	2012	Iran	52 m athletes, of which 22 took part in the study	57 ± 5 y	Osteoarthritis (78.3%)	Nr
Shida et al. [47]	1992	Japan	303 university students (166 m, 137 f)	13–21 y	59.4% (lumbago 23.5%, knee joint injury 13.4%, tenosynovitis, and sprains being the most common injuries)	Age (70% of injuries in high school), practice for more than 5 years, more than 20 h per week, poor training program
Shida et al. [48]	1994	Japan	210 athletes (111 m, 99 f), with 100 taking part in the study	Nr	64.8% (lumbago, 25.1%, shoulder 15.7%, knee 14.1%)	Nr
Shimazaki et al. [20]	2012	Brazil	111 athletes	23 ± 9 y	Injury rate of 0.5 injuries per athlete	International/national players (52.9%) versus regional players (48.8%), most injuries during training
Soligard et al. [49]	2017	Brazil, XXXI Olympic Games, hosted by Rio de Janeiro from 5 to 21 August 2016	11,274 athletes (6185 m, 5089 f) from all disciplines (nr for table tennis)	Nr	0–3%	No risk factors identified
Tin-Oo and Razali [50]	2012	Malaysia	180 athletes	12–27 y	0% (oral injuries)	Nr

Abbreviations: Nr (not reported); y (years).

**Table 3:** List of case reports and case series included in the present scoping review.

Case Report/Case Series	Type of Injury
Avery et al., 1990 [53]	Catastrophic injuries
Copcu, 2004 [51]	Tumor
Dufek et al., 1999 [36]	Orthopedic injuries
Fujiwara, 1995 [52]	Cardiological injuries
Kexue, 1996 [33]	Orthopedic injuries
Le Floch et al., 2010 [6]	Neurological injuries
Li, 1996 [33]	Orthopedic injuries
Nicolini et al., 2014 [32]	Orthopedic injuries
Petschnig et al., 1997 [37]	Orthopedic injuries
Pieper et al., 1993 [31]	Orthopedic injuries
Pintore and Maffulli, 1991 [34]	Orthopedic injuries
Ron et al., 1983 [35]	Orthopedic injuries
Scott and Scott, 1989 [41]	Dermatological injuries
Turk et al., 2008 [54]	Catastrophic injuries

**Table 4:** Risk of bias assessment of the observational studies included in the present scoping review (excluded clinical cases reports/case series).

Authors	Percentage of “Yes”	Interpretation
Anders Niixius et al. [19]	30.0%	High risk of bias
Andrade et al. [40]	33.3%	High risk of bias
Barrell et al. [42]	50.0%	Moderate risk of bias
Chandran [43]	60.0%	Moderate risk of bias
Correa-Mesa and Correa-Morales [21]	11.1%	High risk of bias
Correa-Mesa [7]	55.6%	Moderate risk of bias
de la Torre Combarros et al. [1]	90.0%	Low risk of bias
Di Carlo et al. [23]	33.3%	High risk of bias
Engebretsen et al. [29]	60.0%	Moderate risk of bias
Fernández Córdova and Barrios González [24]	30.0%	High risk of bias
Folorunso et al. [14]	22.2%	High risk of bias
Fong et al. [22]	60.0%	Moderate risk of bias
Hill et al. [39]	60.0%	Moderate risk of bias
Jeffers [44]	40.0%	High risk of bias
Junge et al. [28]	50.0%	Moderate risk of bias
Kelly and Nolan [45]	60.0%	Moderate risk of bias
Kondric et al. [27]	11.1%	High risk of bias
Laoruengthana et al. [38]	40.0%	High risk of bias
Linderoth et al. [30]	11.1%	High risk of bias
Lo et al. [26]	22.2%	High risk of bias
Lovell et al. [46]	70.0%	Moderate risk of bias
Majewski et al. [25]	40.0%	High risk of bias
Rajabi et al. [15]	100.0%	Low risk of bias
Shida et al. [47]	44.4%	High risk of bias
Shida et al. [48]	22.2%	High risk of bias
Shimazaki et al. [20]	60.0%	Moderate risk of bias
Soligard et al. [49]	60.0%	Moderate risk of bias
Tin-Oo and Razali [50]	33.3%	High risk of bias

## Data Availability

All data are available in the main text.
